# Neutrophil-to–high-density lipoprotein cholesterol ratio as a predictor of outcomes after successful endovascular reperfusion in acute ischemic stroke

**DOI:** 10.3389/fneur.2026.1800774

**Published:** 2026-04-15

**Authors:** Ruikai Xu, Mianwei Weng, Qiang Lei, Zhonghua Liu

**Affiliations:** 1 Department of Rehabilitation, Zhongshan People’s Hospital, Zhongshan, Guangdong, China; 2 Department of Tradition Chinese Medicine, Zhongshan People’s Hospital, Zhongshan, Guangdong, China

**Keywords:** acute ischemic stroke, endovascular treatment, high-density lipoprotein cholesterol, neutrophil, rehabilitation

## Abstract

**Background and objective:**

A substantial proportion of patients experience poor functional outcomes despite successful reperfusion after endovascular therapy (EVT) for acute ischemic stroke (AIS). Systemic inflammation and lipid metabolism are thought to contribute to reperfusion-related injury. We aimed to evaluate the association between the neutrophil-to–high-density lipoprotein cholesterol ratio (NHR) and clinical outcomes in AIS patients who achieved successful reperfusion, and to assess its incremental prognostic value beyond established clinical predictors.

**Methods:**

In this single-center retrospective cohort study, 367 AIS patients with successful reperfusion (mTICI 2b–3) after EVT were included. NHR was calculated from baseline blood samples. The primary outcome was poor functional outcome at 90 days (modified Rankin Scale score 3–6). Secondary outcomes included 90-day mortality and symptomatic intracranial hemorrhage (sICH). Associations between NHR and outcomes were examined using logistic regression models with NHR analyzed primarily as a continuous variable, and restricted cubic splines were used to explore potential nonlinearity. Model performance was evaluated by comparing a baseline clinical model with and without NHR using area under the receiver operating characteristic curve (AUC), DeLong tests, calibration metrics, and bootstrap internal validation.

**Results:**

Higher NHR was associated with an increased risk of poor functional outcome after multivariable adjustment (adjusted OR per 1-unit increase, approximately 1.34), indicating a modest effect size. The association with mortality was weaker and showed limited incremental discriminative value beyond baseline clinical predictors. For sICH, the association was attenuated after adjustment and should be interpreted cautiously. Spline analyses suggested possible nonlinearity at higher NHR levels, although confidence intervals widened in these ranges, indicating uncertainty due to sparse data. Adding NHR to the baseline clinical model improved discrimination for poor functional outcome, but the absolute AUC values indicated only moderate predictive performance, and discrimination for mortality and sICH remained weak to modest.

**Conclusion:**

In AIS patients who achieved successful reperfusion after EVT, higher NHR was associated with poorer 90-day functional outcomes, with modest effect sizes and limited incremental prognostic value beyond established clinical markers. NHR should be interpreted as a complementary risk marker rather than a standalone predictive or clinical decision-making tool, and external validation is required before any clinical application.

## 1 Background

As a major contributor to global morbidity and mortality, acute ischemic stroke (AIS) not only leads to a high number of deaths but also leaves a substantial proportion of survivors with significant long-term disabilities, resulting in immense personal suffering and imposing a heavy economic burden on societies (1). Although reperfusion strategies have advanced substantially, overall clinical outcomes remain unsatisfactory (2, 3). Endovascular treatment (EVT) now represents a fundamental therapy for large vessel occlusion stroke, leading to substantially higher rates of successful recanalization (2). Nevertheless, angiographic success does not necessarily translate into meaningful clinical recovery (3).

Functional outcome is commonly assessed using the modified Rankin Scale (mRS) (4). This dissociation between recanalization and recovery likely reflects the combined influence of multiple factors, including infarct core size, collateral circulation, cerebral edema, microvascular no-reflow, and systemic inflammatory responses (3). In clinical practice, it remains difficult to identify which patients will benefit from EVT and which are at increased risk of complications such as symptomatic intracranial hemorrhage (sICH) (1). Importantly, sICH not only negates the benefits of reperfusion but also markedly increases mortality (5). Consequently, there is ongoing interest in biomarkers that capture these complex pathophysiological processes and are associated with outcomes after successful reperfusion.

Increasing evidence highlights the importance of innate immunity and lipid metabolism in reperfusion-related brain injury. Neutrophils rapidly infiltrate ischemic tissue after stroke onset and contribute to blood–brain barrier disruption and microvascular obstruction (6). Multiple clinical observations have confirmed a close relationship between higher neutrophil counts and both expanded cerebral infarct dimensions and impaired neurological recovery (7, 8). In cerebrovascular research, various inflammatory markers—such as neutrophil count, neutrophil-to-lymphocyte ratio, systemic immune-inflammation index, and systemic inflammation response index—have been investigated as prognostic markers, yet findings have been inconsistent and have largely overlooked the counterbalancing protective role of high-density lipoprotein (HDL) (9–12). HDL possesses well-established anti-inflammatory and vasoprotective properties, including suppression of neutrophil activation and oxidative stress, and reduced HDL levels have been consistently linked to increased cardiovascular risk (13, 14).

The neutrophil-to–high-density lipoprotein ratio (NHR) represents an inflammation-related biomarker that integrates both heightened inflammatory activity and diminished lipoprotein-mediated protection, thereby reflecting the balance between proinflammatory burden and HDL-associated vascular defense (15). Elevated NHR has been associated with atherosclerotic cardiovascular events and adverse outcomes after revascularization procedures (16–18). In cerebrovascular disease, several recent studies have examined NHR or related composite indices in EVT-treated populations and have reported associations with stroke severity or outcomes (19, 20). However, these studies have varied in their inclusion criteria, analytic strategies, and handling of reperfusion status and procedural factors, and the incremental prognostic value of NHR beyond established clinical markers has not been systematically evaluated (19, 20).

In this context, the present study focused on patients with AIS who achieved successful reperfusion after EVT (mTICI 2b–3) and examined the association between NHR and subsequent clinical outcomes, including 90-day functional outcome, mortality, and sICH. Rather than proposing NHR as a clinical decision-making tool, our aim was to assess whether NHR is associated with outcomes in this specific setting and whether it provides incremental prognostic information beyond established predictors such as admission NIHSS score and ASPECTS. We hypothesized that higher NHR levels would be associated with poorer outcomes after successful reperfusion, reflecting a greater inflammatory–metabolic imbalance in the acute phase.

## 2 Methods

### 2.1 Study population

This retrospective cohort study was approved by the Institutional Review Board of Zhongshan People’s Hospital and conducted in accordance with the Declaration of Helsinki. The requirement for informed consent was waived because the study used previously collected medical records. Between February 2022 and May 2025, 367 consecutive AIS patients who were treated with EVT at our institution were retrospectively reviewed. Successful reperfusion was confirmed by postoperative digital subtraction angiography (DSA) and defined as a modified Thrombolysis in Cerebral Infarction (mTICI) score of 2b or 3 (21). The mTICI grade was assessed by two experienced clinicians.

### 2.2 Inclusion and exclusion criteria

**Figure 1:**
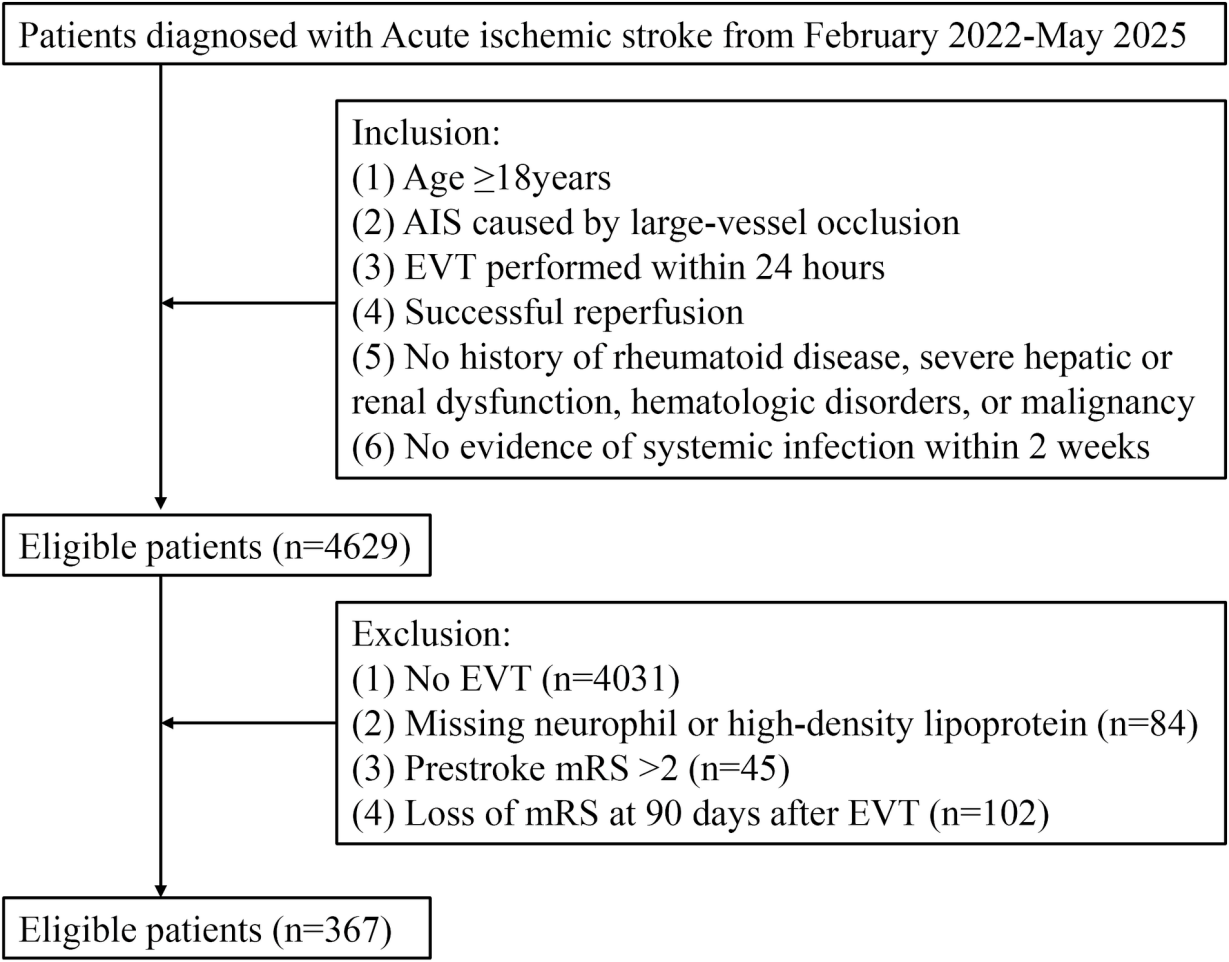
Flow chart of participants selection.

Patients were eligible if they met the following criteria: (1) age ≥18 years; (2) AIS caused by large-vessel occlusion; (3) EVT performed within 24 h of symptom onset; (4) successful reperfusion confirmed by postoperative DSA; (5) no history of rheumatoid disease, severe hepatic or renal dysfunction, hematologic disorders, or malignancy; and (6) no evidence of systemic infection within two weeks prior to stroke onset. Patients were excluded if they: (1) underwent diagnostic cerebral angiography without EVT; (2) lacked baseline neutrophil or high-density lipoprotein measurements; (3) had a pre-stroke modified Rankin Scale (mRS) score >2; or (4) had missing 90-day follow-up outcome data (22) (Figure 1).

### 2.3 Data collection

Demographic characteristics (age and sex), lifestyle factors (smoking and alcohol use), admission systolic and diastolic blood pressure, National Institutes of Health Stroke Scale (NIHSS) score, and Alberta Stroke Program Early CT Score (ASPECTS) were extracted from the electronic medical record system. Medical history included prior intravenous thrombolysis, previous stroke, hypertension, diabetes mellitus, hyperlipidemia, coronary artery disease, and atrial fibrillation (23). Stroke-related variables comprised anterior or posterior circulation involvement, occlusion site, and Trial of Org 10,172 in Acute Stroke Treatment (TOAST) etiological subtype (23). Procedural variables included time from symptom onset to hospital admission, imaging, and groin puncture; collateral circulation grade (0–1, poor; 2, moderate; 3–4, good); whether more than three thrombectomy passes were performed; and the use of intra-arterial thrombolysis, balloon angioplasty, stent implantation or retrieval, catheter aspiration, and tirofiban (24, 25). Complications included pneumonia and symptomatic intracranial hemorrhage (sICH), the latter defined by the Heidelberg Bleeding Classification as intracranial hemorrhage linked to neurological worsening. This was shown as a > 4-point increase in total NIHSS score, a ≥ 2-point rise in any NIHSS item, or clinical decline requiring major intervention (4, 26).

### 2.4 Calculation of NHR

The neutrophil-to–high-density lipoprotein ratio (NHR) was calculated as the peripheral neutrophil count (×10^9^/L) divided by the high-density lipoprotein cholesterol concentration (mmol/L) (18).

### 2.5 Clinical outcomes

The primary outcome was functional outcome at 90 days after EVT (4). Poor functional outcome was defined as an mRS score of 3–6, whereas favorable outcome was defined as an mRS score of 0–2. Secondary outcomes included all-cause mortality within 90 days and the occurrence of sICH (4). Outcome data were obtained through review of inpatient and outpatient records and structured telephone follow-up.

### 2.6 Statistical analysis

Continuous variables are presented as median (interquartile range, IQR) or mean ± standard deviation (SD), as appropriate, and categorical variables as counts (percentages). Comparisons across NHR tertiles were performed using the Kruskal–Wallis test or one-way analysis of variance (ANOVA) for continuous variables and the χ^2^ test or Fisher’s exact test for categorical variables, as appropriate. The primary exposure of interest was the NHR. NHR was analyzed primarily as a continuous variable. For descriptive purposes, NHR was additionally categorized into tertiles; however, categorical analyses were considered supplementary and were not used for primary inference to avoid information loss and arbitrary threshold effects. Associations between NHR and clinical outcomes, including 90-day poor functional outcome, 90-day mortality, and sICH, were assessed using logistic regression models. Effect estimates are reported as odds ratios (ORs) with 95% confidence intervals (CIs) per 1-unit increase in NHR. Three hierarchical models were prespecified: (i) an unadjusted (crude) model including NHR only; (ii) a clinically motivated core-adjusted model including age, sex, admission NIHSS score, ASPECTS, and onset-to-groin puncture time; and (iii) a stepwise expanded model additionally incorporating stroke characteristics and key procedural variables as sensitivity analyses to evaluate robustness under broader adjustment. Given the number of covariates relative to the number of outcome events, events-per-variable (EPV) was calculated for each outcome and model, and the core-adjusted model was prioritized for primary inference. Multicollinearity was assessed using variance inflation factors (VIF). Model stability was further examined using bootstrap resampling (≥500 iterations) and penalized logistic regression as supplementary analyses. Potential nonlinear relationships between NHR and each outcome were evaluated using restricted cubic spline (RCS) functions with three knots placed at the 10th, 50th, and 90th percentiles of the NHR distribution. The presence of nonlinearity was assessed using likelihood ratio tests comparing models with and without nonlinear spline terms. Spline curves were presented as adjusted risk estimates across the observed range of NHR. To assess the incremental prognostic value of NHR, we constructed a baseline clinical model including age, admission NIHSS score, and ASPECTS, and an extended model additionally incorporating NHR. Discrimination was quantified using the area under the receiver operating characteristic curve (AUC), and differences in AUC between models were compared using the DeLong test. Calibration was evaluated using the Brier score and calibration plots. Internal validation was performed using bootstrap resampling to estimate optimism-corrected AUC and calibration slope. Optimal NHR cut-offs were derived using Youden’s index for descriptive purposes only. These thresholds were considered exploratory and dataset-specific and were not interpreted as clinically actionable without external validation. All statistical tests were two-sided, and a *p* value < 0.05 was considered statistically significant. Analyses were performed using Python (statsmodels, scikit-learn, and related packages).

## 3 Results

### 3.1 Baseline characteristics

A total of 367 patients with large vessel occlusion acute ischemic stroke who achieved successful reperfusion after endovascular therapy were included in the final analysis. Patients were stratified into tertiles according to NHR (T1, *n* = 123; T2, *n* = 121; T3, *n* = 123) (Table 1). The overall median age was 61.0 years (IQR, 54.0–71.0), and 73.3% of patients were male. Several baseline characteristics differed across NHR tertiles. Patients in higher NHR tertiles were younger (median age: 63.0, 63.0, and 59.0 years from T1 to T3; *p* = 0.013) and more frequently smokers (25.2, 16.5, and 31.7%; *p* = 0.022). Admission stroke severity differed significantly across groups, with higher NIHSS scores observed in T2 and T3 compared with T1 (median NIHSS: 7.0, 12.0, and 11.0; *p* < 0.001), whereas ASPECTS did not differ significantly (*p* = 0.427). Regarding stroke subtype and occlusion site, most distributions were comparable, although basilar artery occlusion was more frequent in the highest NHR tertile (6.5, 9.1, and 17.9%; *p* = 0.012). The prevalence of major vascular risk factors and prior treatments, including hypertension, diabetes, hyperlipidemia, coronary artery disease, atrial fibrillation, and intravenous thrombolysis, did not differ significantly between groups. Postoperative complications showed significant gradients across tertiles. The incidence of pneumonia increased from 16.3% in T1 to 30.1% in T3 (*p* = 0.035), and the incidence of symptomatic intracranial hemorrhage (sICH) increased markedly across tertiles (8.9, 7.4, and 26.8%; *p* < 0.001). Among procedural time metrics, onset-to-admission and onset-to-imaging times differed significantly across groups (*p* = 0.022 and *p* = 0.033, respectively), whereas onset-to-groin puncture time did not (*p* = 0.275). Use of catheter aspiration was more frequent in higher tertiles (36.6, 48.8, and 54.5%; *p* = 0.016), while other procedural variables were broadly comparable. Neutrophil counts increased and HDL levels decreased across increasing NHR tertiles (both *p* < 0.001), resulting in progressively higher NHR values (median NHR: 3.51, 5.80, and 9.09; *p* < 0.001). Clinical outcomes also differed significantly: median 90-day mRS scores increased across tertiles (2.0, 3.0, and 4.0; *p* < 0.001), and the proportions of poor functional outcome (30.1, 51.2, and 79.7%; *p* < 0.001) and 90-day mortality (5.7, 7.4, and 16.3%; *p* = 0.012) were highest in the upper NHR tertile (Table 1).

**Table 1:** Basic characteristics among groups categorized by the NHR index.

	Total (*n* = 367)	T1 (*n* = 123)	T2 (*n* = 121)	T3 (*n* = 123)	*p* value
Demographics					
Sex, Male, *n* (%)	269 (73.30%)	83 (67.48%)	89 (73.55%)	97 (78.86%)	0.130
Age (years)	61.00 (54.00, 71.00)	63.00 (55.50, 73.00)	63.00 (54.00, 71.00)	59.00 (52.00, 68.00)	0.013
Smoking, *n* (%)	90 (24.52%)	31 (25.20%)	20 (16.53%)	39 (31.71%)	0.022
Drinking, *n* (%)	45 (12.26%)	19 (15.45%)	12 (9.92%)	14 (11.38%)	0.393
Systolic blood pressure (mmHg)	148.00 (133.00, 162.00)	145.00 (133.00, 161.00)	148.00 (132.00, 164.00)	151.00 (136.00, 161.50)	0.723
Diastolic blood pressure (mmHg)	89.00 (79.00, 97.00)	87.00 (78.00, 96.00)	89.00 (80.00, 100.00)	89.00 (80.00, 96.00)	0.226
NIHSS on admission	10.00 (5.00, 16.00)	7.00 (3.50, 12.50)	12.00 (7.00, 17.00)	11.00 (7.00, 16.00)	<0.001
ASPECT	9.00 (7.00, 10.00)	9.00 (7.00, 10.00)	9.00 (7.00, 10.00)	9.00 (7.00, 10.00)	0.427
Medical history, *n* (%)					
Intravenous thrombolysis	59 (16.08%)	25 (20.33%)	15 (12.40%)	19 (15.45%)	0.235
History of stroke	53 (14.44%)	22 (17.89%)	16 (13.22%)	15 (12.20%)	0.401
Hypertension	252 (68.66%)	81 (65.85%)	83 (68.60%)	88 (71.54%)	0.629
Diabetes	108 (29.43%)	36 (29.27%)	31 (25.62%)	41 (33.33%)	0.417
Hyperlipidemia	135 (36.78%)	47 (38.21%)	47 (38.84%)	41 (33.33%)	0.619
Coronary artery disease	29 (7.90%)	11 (8.94%)	11 (9.09%)	7 (5.69%)	0.537
Atrial fibrillation	93 (25.34%)	36 (29.27%)	33 (27.27%)	24 (19.51%)	0.178
Procedural time (min)					
Time from onset to admission	195.00 (107.50, 358.00)	136.00 (72.50, 350.50)	197.00 (123.00, 335.00)	225.00 (122.00, 363.50)	0.022
Time from onset to image	214.00 (121.50, 379.50)	175.00 (81.00, 368.50)	216.00 (131.00, 354.00)	235.00 (131.00, 410.50)	0.033
Time from onset to groin puncture	330.00 (228.00, 539.00)	325.00 (205.50, 530.00)	321.00 (230.00, 490.00)	351.00 (242.50, 555.50)	0.275
Site of occlusion, *n* (%)					
ICA	104 (28.34%)	33 (26.83%)	33 (27.27%)	38 (30.89%)	0.740
MCA	181 (49.32%)	66 (53.66%)	63 (52.07%)	52 (42.28%)	0.155
ICA+MCA	13 (3.54%)	3 (2.44%)	6 (4.96%)	4 (3.25%)	0.555
Basilar	41 (11.17%)	8 (6.50%)	11 (9.09%)	22 (17.89%)	0.012
Others	28 (7.63%)	13 (10.57%)	8 (6.61%)	7 (5.69%)	0.310
Type of TOAST, *n* (%)					
Large artery atherosclerosis	279 (76.02%)	92 (74.80%)	91 (75.21%)	96 (78.05%)	0.810
Cardioembolism	77 (20.98%)	28 (22.76%)	27 (22.31%)	22 (17.89%)	0.584
Others	11 (3.00%)	3 (2.44%)	3 (2.48%)	5 (4.07%)	0.696
Anterior circulation infarction, *n* (%)	309 (84.20%)	109 (88.62%)	104 (85.95%)	96 (78.05%)	0.061
Collateral circulation status	2.00 (1.00, 3.00)	2.00 (2.00, 3.00)	2.00 (1.00, 3.00)	2.00 (1.00, 3.00)	0.194
Operation strategy					
Number of passes >3 times, *n* (%)	91 (24.80%)	27 (21.95%)	28 (23.14%)	36 (29.27%)	0.362
Arterial thrombolysis, *n* (%)	39 (10.63%)	14 (11.38%)	10 (8.26%)	15 (12.20%)	0.576
Balloon dilatation, *n* (%)	171 (46.59%)	56 (45.53%)	51 (42.15%)	64 (52.03%)	0.290
Stent implantation, *n* (%)	59 (16.08%)	20 (16.26%)	18 (14.88%)	21 (17.07%)	0.895
Stent removal, *n* (%)	252 (68.66%)	81 (65.85%)	88 (72.73%)	83 (67.48%)	0.482
Catheter aspiration, *n* (%)	171 (46.59%)	45 (36.59%)	59 (48.76%)	67 (54.47%)	0.016
Tirofiban treatment, *n* (%)	244 (66.49%)	73 (59.35%)	87 (71.90%)	84 (68.29%)	0.101
Postoperative complications, *n* (%)					
Pneumonia	84 (22.89%)	20 (16.26%)	27 (22.31%)	37 (30.08%)	0.035
Symptomatic ICH	53 (14.44%)	11 (8.94%)	9 (7.44%)	33 (26.83%)	<0.001
Neutrophil count	6.71 (5.04, 9.12)	4.49 (3.70, 5.58)	6.71 (5.72, 7.78)	9.91 (8.12, 12.66)	<0.001
HDL level	1.16 (1.00, 1.41)	1.36 (1.14, 1.62)	1.15 (1.00, 1.37)	1.08 (0.92, 1.21)	<0.001
NHR	5.80 (4.17, 7.94)	3.51 (2.97, 4.17)	5.80 (5.21, 6.24)	9.09 (7.94, 11.80)	<0.001
mRS at 90 days	3.00 (2.00, 4.00)	2.00 (1.50, 3.00)	3.00 (2.00, 4.00)	4.00 (3.00, 5.00)	<0.001
Poor outcome at 90 days, *n* (%)	197 (53.68%)	37 (30.08%)	62 (51.24%)	98 (79.67%)	<0.001
Mortality at 90 days, *n* (%)	36 (9.81%)	7 (5.69%)	9 (7.44%)	20 (16.26%)	0.012

ASPECTS, Alberta Stroke Program Early Computed Tomography Score; ICA, Internal carotid artery; MCA, middle cerebral artery; mRS, modified Rankin Scale; NHR, neutrophil-to–high-density lipoprotein cholesterol ratio; NIHSS, National Institutes of Health Stroke Scale; TOAST, Trial of Org10172 in Acute Stroke Treatment.

Given these substantial baseline imbalances in stroke severity, occlusion site, postoperative complications, and time-to-treatment metrics, all primary inferences were therefore based on continuous NHR with prespecified multivariable adjustment.

### 3.2 Events per variable and model feasibility

For 90-day poor functional outcome, there were 197 events among 367 patients, yielding an EPV of 32.83 for the core-adjusted model and 8.21 for the expanded model. For 90-day mortality (36 events), the EPV was 6.00 for the core-adjusted model and 1.50 for the expanded model. For sICH (53 events), the EPV was 8.83 for the core-adjusted model and 2.21 for the expanded model. Given these EPV profiles, the core-adjusted model was prioritized for primary inference, whereas the expanded models were considered sensitivity analyses to examine robustness under broader adjustment.

### 3.3 Association between continuous NHR and clinical outcomes

When NHR was analyzed as a continuous variable (per 1-unit increase), higher NHR was associated with a modest but consistent increase in the risk of adverse clinical outcomes (Table 2). For 90-day poor functional outcome, NHR was associated with increased risk in the crude model (OR, 1.38; 95% CI, 1.25–1.52; *p* < 0.001), and the association remained after core adjustment for age, sex, admission NIHSS score, ASPECTS, and onset-to-groin puncture time (OR, 1.34; 95% CI, 1.21–1.48; *p* < 0.001) as well as in the stepwise expanded model (OR, 1.36; 95% CI, 1.22–1.51; *p* < 0.001). For 90-day mortality, NHR was associated with increased risk in the crude model (OR, 1.13; 95% CI, 1.05–1.21; *p* < 0.001) and remained statistically significant in the core-adjusted (OR, 1.13; 95% CI, 1.05–1.21; *p* = 0.001) and expanded models (OR, 1.11; 95% CI, 1.02–1.20; *p* = 0.011). However, as shown below, the incremental discriminative gain for mortality was limited, indicating a weaker and less robust signal than that observed for poor functional outcome. For sICH, higher NHR was associated with increased odds in the crude model (OR, 1.10; 95% CI, 1.03–1.16; *p* = 0.003) and after core adjustment (OR, 1.09; 95% CI, 1.03–1.16; *p* = 0.004). In the expanded model, the association was attenuated and of borderline statistical significance (OR, 1.07; 95% CI, 1.00–1.14; *p* = 0.047) (Table 2). Overall, the effect size for sICH was modest and became weaker with broader adjustment, and this association should therefore be interpreted cautiously. Tertile-based comparisons are presented for descriptive purposes only and are not used for primary inference, as categorization may amplify effect estimates and introduce arbitrary thresholds.

**Table 2:** Association between continuous NHR and clinical outcomes.

NHR	Crude model		Core-adjusted model		Expanded model	
	OR (95%CI)	*p* value	OR (95%CI)	*p* value	OR (95%CI)	*p* value
Poor outcome at 90 days						
Continuous	1.38 (1.25, 1.52)	<0.001	1.34 (1.21, 1.48)	<0.001	1.36 (1.22, 1.51)	<0.001
Mortality at 90 days						
Continuous	1.13 (1.05, 1.21)	<0.001	1.13 (1.05, 1.21)	0.001	1.11 (1.02, 1.20)	0.011
sICH						
Continuous	1.10 (1.03, 1.16)	0.003	1.09 (1.03, 1.16)	0.004	1.07 (1.00, 1.14)	0.047

Model: (i) crude model including NHR only; (ii) core-adjusted model including age, sex, admission NIHSS score, ASPECTS, and onset-to-groin puncture time; and (iii) expanded model additionally incorporating stroke characteristics and key procedural variables including age, sex, admission NIHSS score, ASPECTS, anterior circulation infarction, collateral circulation status, history of stroke, hypertension, diabetes, hyperlipidemia, coronary artery disease, atrial fibrillation, intravenous thrombolysis, pneumonia, onset-to-admission time, onset-to-image time, onset-to-groin puncture time, number of passes >3 times, arterial thrombolysis, balloon dilatation, stent implantation, stent removal, and tirofiban treatment.

NHR, neutrophil-to–high-density lipoprotein cholesterol ratio; sICH, Symptomatic intracranial hemorrhage.

### 3.4 Multicollinearity and model stability

Multicollinearity was assessed using VIF (Supplementary Table S1). In the core-adjusted models, VIF values were generally acceptable, with NHR showing low-to-moderate collinearity. In the expanded models, higher VIFs were observed for some covariates, consistent with correlations among closely related clinical and procedural metrics. Model stability was further evaluated using bootstrap resampling and penalized logistic regression. Bootstrap estimates for the NHR effect were consistent with the primary model point estimates, with mean ORs (95% percentile intervals) of 1.35 (1.17–1.61) for 90-day poor functional outcome, 1.13 (1.07–1.22) for 90-day mortality, and 1.11 (1.04–1.24) for sICH. Penalized regression yielded similar NHR effect estimates (ridge ORs: 1.34, 1.09, and 1.05 for poor functional outcome, mortality, and sICH, respectively), supporting the overall robustness of the findings to alternative modeling strategies.

### 3.5 Nonlinear association between NHR and outcomes

**Figure 2:**
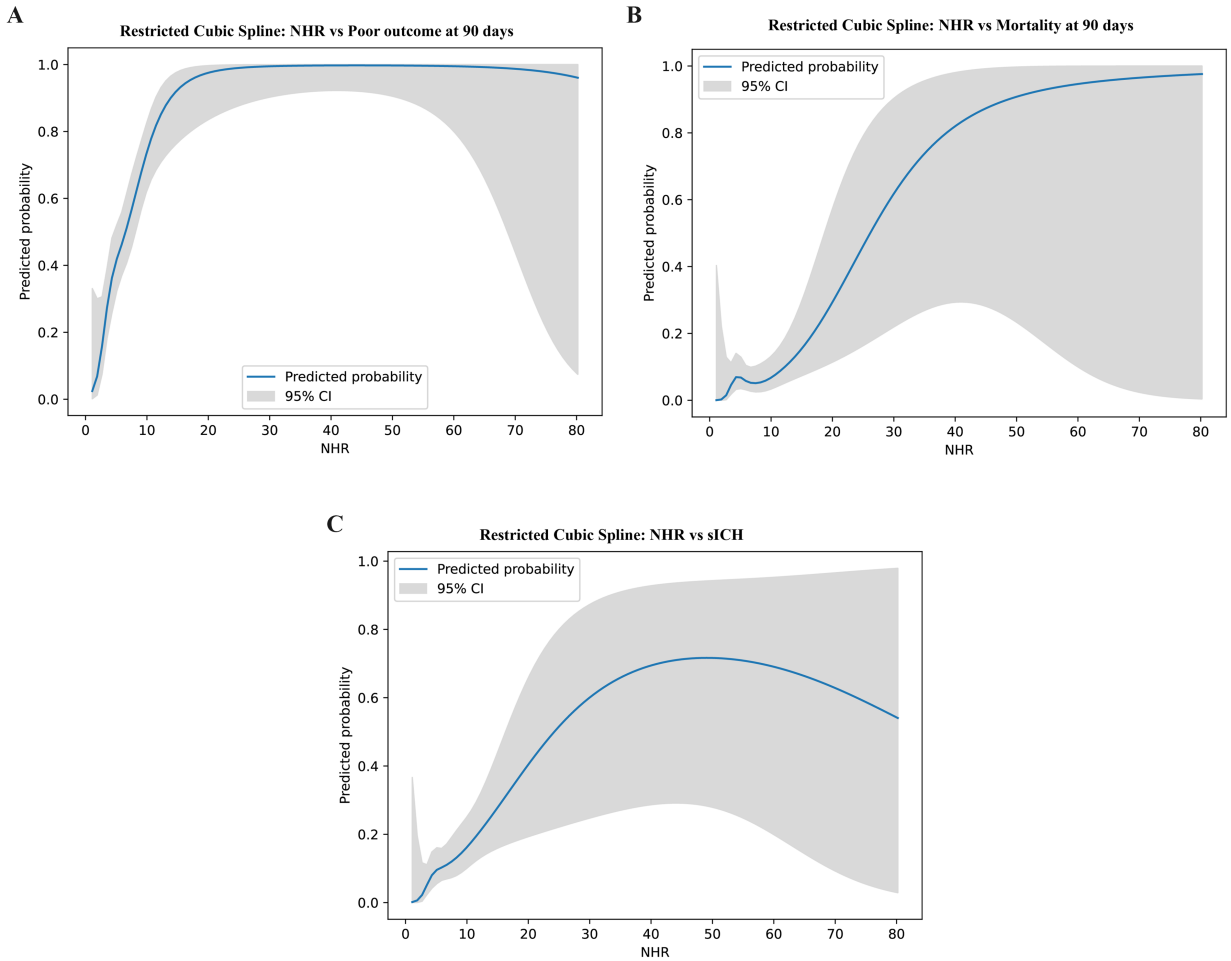
**(A)** Restricted cubic spline showing correlation between NHR levels and poor outcome. **(B)** Restricted cubic spline showing correlation between NHR levels and mortality. **(C)** Restricted cubic spline showing correlation between NHR levels and sICH.

RCS models with three knots placed at the 10th, 50th, and 90th percentiles of the NHR distribution were fitted in the core-adjusted framework to explore potential nonlinearity. For 90-day poor functional outcome, the adjusted spline curve suggested a monotonic increase in predicted risk with higher NHR values, with a borderline improvement in fit compared with a linear NHR term (likelihood ratio test [LRT] *p* = 0.064) (Figure 2A). For 90-day mortality, no clear nonlinear pattern was observed (LRT *p* = 0.367) (Figure 2B). For sICH, the spline analysis suggested a nonlinear association (LRT *p* = 0.043), with the risk increasing more prominently at higher NHR levels (Figure 2C). Notably, confidence intervals widened at higher NHR levels, indicating increasing uncertainty in regions with sparse observations; therefore, apparent inflection patterns should not be interpreted as definitive biological thresholds.

### 3.6 Incremental prognostic value of NHR beyond baseline clinical variables

**Figure 3:**
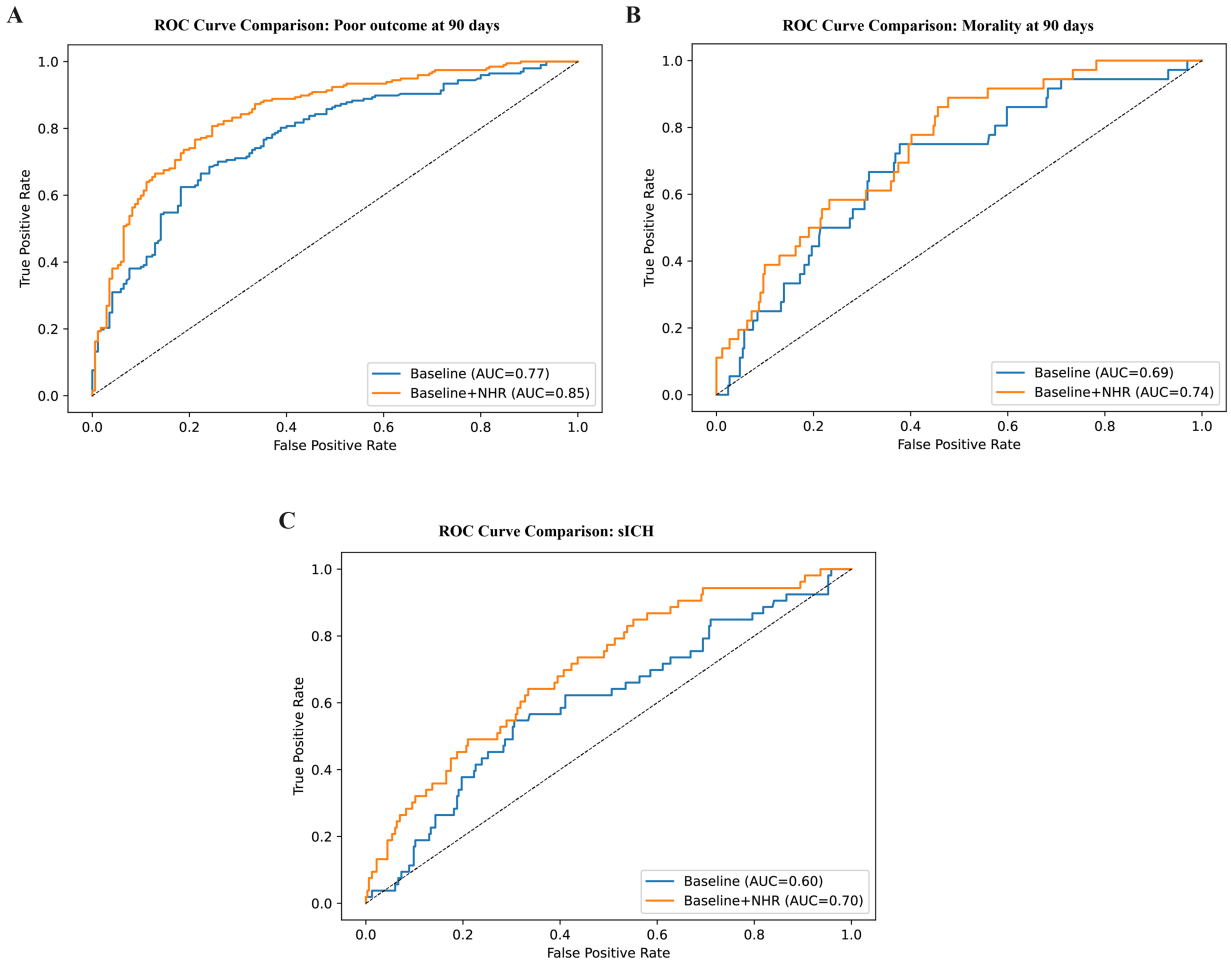
**(A)** ROC curve comparison showing incremental prognostic value of NHR beyond baseline clinical variables for poor outcome. **(B)** ROC curve comparison showing incremental prognostic value of NHR beyond baseline clinical variables for mortality. **(C)** ROC curve comparison showing incremental prognostic value of NHR beyond baseline clinical variables for sICH.

We compared a baseline clinical model including age, admission NIHSS score, and ASPECTS with an extended model additionally incorporating NHR. For 90-day poor functional outcome, adding NHR improved discrimination, with AUC increasing from 0.773 to 0.845 (ΔAUC = 0.072; DeLong *p* = 0.000020) (Figure 3A). For 90-day mortality, AUC increased from 0.687 to 0.745 (ΔAUC = 0.058), but the difference was not statistically significant by DeLong testing (*p* = 0.163) (Figure 3B). For sICH, adding NHR improved AUC from 0.601 to 0.701 (ΔAUC = 0.099; DeLong *p* = 0.004081) (Figure 3C). Thus, although statistically significant improvements in discrimination were observed for some endpoints, the magnitude of these gains was clinically modest. Calibration was evaluated using calibration plots and Brier scores (Supplementary Figures S1A–C). The models including NHR showed acceptable overall calibration, with Brier scores of 0.159 for poor functional outcome, 0.079 for mortality, and 0.116 for sICH. Bootstrap internal validation suggested limited optimism, with optimism-corrected AUCs of 0.839, 0.721, and 0.673 for poor functional outcome, mortality, and sICH, respectively, in the baseline+NHR models. However, the absolute AUC values indicate only moderate discrimination for poor functional outcome and weak-to-modest discrimination for mortality and sICH, underscoring that NHR alone is insufficient for high-accuracy individual-level prediction.

### 3.7 Exploratory NHR cut-off values

For descriptive purposes only, outcome-specific NHR cut-offs were derived using Youden’s index (Supplementary Table S2). The optimal cut-off was 5.80 for 90-day poor functional outcome (sensitivity 0.68, specificity 0.69), 8.62 for 90-day mortality (sensitivity 0.44, specificity 0.83), and 7.31 for sICH (sensitivity 0.62, specificity 0.73). These thresholds are exploratory and dataset-specific and should not be interpreted as clinically actionable without external validation.

## 4 Discussion

A substantial proportion of patients experience poor functional recovery despite successful reperfusion after EVT. In this study, we evaluated the association between NHR and outcomes in this setting. We found that higher NHR was associated with worse functional outcomes after adjustment for major clinical confounders; however, the observed effect sizes were modest, and the overall discriminative performance remained moderate. These findings indicate that NHR should be interpreted as a complementary risk marker rather than a standalone predictive tool, and the present results require external validation before any clinical application can be considered.

Neutrophils are among the earliest immune responders following acute ischemic stroke and play a pivotal role throughout the ischemia–reperfusion cascade (27). After stroke onset, neutrophil counts rise rapidly, reflecting enhanced mobilization from the bone marrow and spleen as well as reduced apoptotic clearance (6). Neutrophils upregulate adhesion molecules within minutes, accumulate within the cerebral microvasculature within hours, and progressively infiltrate brain parenchyma over the ensuing 24–48 h (28–30). Within the ischemic penumbra and perivascular spaces, neutrophils interact with endothelial cells and platelets, releasing reactive oxygen species, proteolytic enzymes, proinflammatory mediators, and neutrophil extracellular traps (6). These processes disrupt the blood–brain barrier, promote microvascular thrombosis, and exacerbate cerebral edema and neuronal injury (6). Beyond acute injury, neutrophils also contribute to atherogenesis and plaque destabilization by mediating oxidative modification of low-density lipoproteins, promoting foam cell formation, and degrading fibrous caps (31–34). Nevertheless, although these mechanisms are biologically plausible, the magnitude of the association observed in our cohort was modest, suggesting that NHR captures only a limited component of the complex pathophysiology underlying post-EVT outcomes. Similar patterns have been reported in previous studies suggesting that their effects may be partially mediated through plaque burden, microvascular no-reflow, and reperfusion-related injury (7, 35–37).

Consistent with prior EVT-related studies, our results support the prognostic relevance of neutrophil-related inflammatory activity in ischemic stroke. Previous investigations have shown that neutrophil counts and neutrophil-derived indices are associated with unfavorable outcomes after endovascular treatment (8, 22, 35, 38). In our cohort, NHR showed a statistically significant association with 90-day poor functional outcome when modeled as a continuous variable, even after adjustment for demographic factors, stroke severity, imaging characteristics, and procedural variables. However, the effect size per unit increase in NHR was modest, indicating that neutrophil-dominant inflammation likely represents one of several contributing factors rather than a dominant determinant of neurological recovery after successful recanalization.

Spline analyses suggested a steeper increase in the risk of poor functional outcome at higher NHR levels (39), whereas the association with mortality was comparatively weaker. In particular, although NHR was associated with mortality when modeled continuously, the incremental discriminative gain over established clinical predictors was not statistically significant, indicating limited robustness of the mortality signal. These findings imply that neutrophil-related inflammation may exert a greater influence on functional impairment than on short-term mortality, which is driven by multiple competing mechanisms. Importantly, confidence intervals widened at higher NHR levels, reflecting data sparsity in the extremes; therefore, apparent inflection patterns should not be interpreted as evidence of biologically meaningful thresholds and require external validation before any clinical inference can be made.

In contrast to the pro-inflammatory effects of neutrophils, high-density lipoprotein (HDL) is widely recognized for its multifaceted vascular protective properties, including reverse cholesterol transport as well as anti-inflammatory, antioxidant, and antithrombotic actions (14). HDL promotes the efflux of excess cholesterol from peripheral tissues and the vascular wall to the liver for metabolism, thereby slowing atherosclerotic plaque formation (40). Beyond lipid transport, HDL mitigates chronic vascular injury by suppressing inflammatory mediator release, reducing vascular wall inflammation, and preserving endothelial integrity (40). Moreover, HDL-associated antioxidant enzymes facilitate the clearance of reactive oxygen species and inhibit the oxidative modification of low-density lipoprotein into oxidized LDL, thereby interrupting atherogenesis at an early stage (41, 42). At the coagulation–fibrinolysis interface, HDL limits pathological thrombosis by restraining platelet overactivation and maintaining hemostatic balance, ultimately lowering the risk of ischemic cerebrovascular events (14). Consistent evidence links low HDL-C levels to an increased atherosclerotic burden and a higher incidence of heart disease and stroke (14, 43). Several stroke cohort studies have further indicated that low HDL levels are linked to an increased risk of ischemic stroke occurrence and recurrence, although data specific to EVT-treated populations remain relatively sparse (14).

In our study, HDL constituted the denominator of the NHR index; under comparable inflammatory conditions, lower HDL levels translated into higher NHR values. A reduction in HDL implies diminished anti-inflammatory and antioxidant protection, an effect that may be further amplified in the presence of heightened neutrophil activation (44, 45). However, the modest effect sizes observed in our analyses suggest that this balance captures only a limited aspect of the overall injury and recovery processes after EVT.

Consistent with this interpretation, we found that NHR demonstrated only moderate discriminative performance for 90-day poor functional outcome and weak-to-modest performance for mortality and sICH. Moreover, the incremental gain in discrimination beyond established predictors such as admission NIHSS score and ASPECTS was statistically significant only for some endpoints and clinically modest in magnitude. This indicates that NHR provides limited incremental prognostic information beyond well-established severity-based markers and should be viewed as a supplementary rather than a replacement variable in risk assessment (14).

With respect to symptomatic intracranial hemorrhage, NHR was associated with hemorrhagic risk in unadjusted analyses but the association was attenuated with multivariable adjustment, indicating potential confounding by baseline clinical and treatment-related factors. Although spline analyses suggested a possible increase in risk at higher NHR levels, the widening confidence intervals in these ranges underscore substantial uncertainty. Therefore, our findings do not support the use of NHR as an independent hemorrhagic risk stratifier. This pattern suggests that, within a certain range, inflammatory activity and HDL-mediated vascular protection may remain in relative equilibrium; however, once neutrophil-driven inflammation exceeds the protective capacity of HDL, blood–brain barrier stability may be substantially compromised, thereby increasing susceptibility to hemorrhagic complications (13, 36, 44). This interpretation aligns with the findings of Xin Jiang et al., who reported an inverse association between NHR and ineffective reperfusion at lower levels but a positive association at higher levels (46). From a pathophysiological standpoint, these observations support the potential utility of NHR in post-EVT hemorrhagic risk assessment and help explain the borderline or trend-level associations observed in multivariable analyses (47).

The NHR integrates two biologically opposing processes—neutrophil-driven inflammation and HDL-mediated vascular protection—and thus reflects a composite systemic inflammatory–metabolic milieu after reperfusion (14, 27). While this “balance-oriented” perspective is conceptually appealing, our results indicate that NHR is associated with outcomes with modest effect sizes and limited incremental predictive value. Accordingly, NHR should be interpreted as a complementary risk marker rather than a clinically actionable stratification tool. Mechanistically, a high NHR state may reflect intensified neutrophil–endothelial interactions, increased microvascular obstruction, and enhanced formation of neutrophil extracellular traps, coupled with diminished HDL-mediated antioxidant and anti-inflammatory protection (3, 44). This imbalance may compromise microcirculatory reperfusion efficiency early after recanalization and aggravate blood–brain barrier disruption at later stages, ultimately restricting neurological recovery and, under certain conditions, predisposing patients to hemorrhagic complications (36, 48).

Finally, although NHR is derived from routine laboratory tests and is inexpensive and readily available, our data do not support its use to guide individualized peri-procedural management or rehabilitation strategies at this stage. Future studies incorporating external validation and formal clinical utility or decision-analytic frameworks are required to determine whether adding NHR to existing models can translate into meaningful improvements in clinical decision-making.

### 4.1 Limitations

Several limitations of this study should be acknowledged. First, this was a retrospective, single-center analysis, which introduces potential selection bias and limits generalizability; therefore, larger multicenter prospective studies are required to confirm the robustness of our findings (49). Second, although we performed internal validation using bootstrap resampling and assessed model calibration, no external validation cohort was available, and internal performance alone is insufficient to support clinical implementation. Third, despite prespecified multivariable adjustment, residual confounding cannot be excluded, particularly with respect to stroke severity, systemic stress, and treatment delays. Fourth, although NHR was primarily analyzed as a continuous variable, tertile-based categorization was used for descriptive purposes, which may amplify effect estimates and introduce arbitrary thresholds. Fifth, while we evaluated the incremental discriminative value of NHR using changes in AUC and DeLong testing, we did not perform formal reclassification or decision-analytic analyses (e.g., net reclassification improvement or decision curve analysis), and therefore the clinical utility of adding NHR to existing models remains uncertain. Finally, only baseline NHR values were analyzed, and temporal changes in NHR as well as functional properties of HDL were not assessed (50), limiting insight into dynamic inflammatory and lipid-related responses (51).

## 5 Conclusion

In patients with acute ischemic stroke who achieved successful reperfusion after EVT, higher NHR was associated with poorer 90-day functional outcomes after adjustment for major clinical confounders. However, the observed effect sizes were modest, and the incremental predictive value beyond established markers such as admission NIHSS score and ASPECTS was limited. These findings indicate that NHR should be interpreted as a complementary risk marker reflecting the inflammatory–metabolic milieu rather than a practical risk stratification tool or a basis for clinical decision-making.

## Data Availability

The data that support the findings of this study are not publicly available due to restrictions imposed by the ethical approval and the privacy of patient medical records. However, de-identified data may be made available to qualified researchers upon reasonable request from the corresponding author, subject to a formal data sharing agreement and approval by the Institutional Review Board of Zhongshan People’s Hospital. Requests to access these datasets should be directed to ZL, zhonghua_reha@163.com.

## Supplementary material

SUPPLEMENTARY FIGURE S1 **(A)** Calibration plot of NHR for poor outcome. **(B)** Calibration plot of NHR for mortality. **(C)** Calibration plot of NHR for sICH.
