# Supplementary figures and images for: Neutrophil-to–high-density lipoprotein cholesterol ratio as a predictor of outcomes after successful endovascular reperfusion in acute ischemic stroke
Source: Front Neurol. 2026 Apr 15;17:1800774. doi: 10.3389/fneur.2026.1800774 (PMC13125049; doi:10.3389/fneur.2026.1800774)

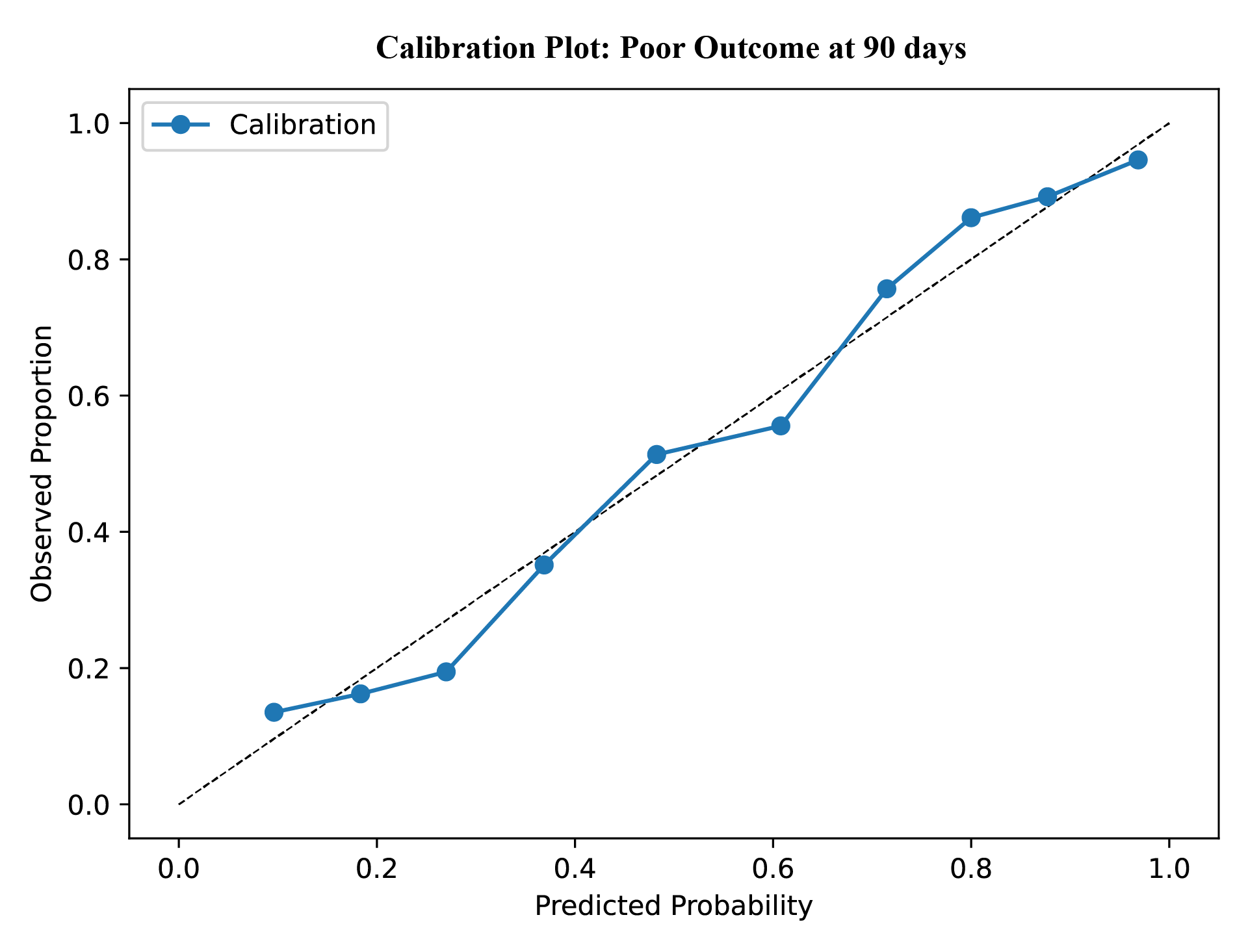

Supplement: SUPPLEMENTARY FIGURE S1 — (A) Calibration plot of NHR for poor outcome. (B) Calibration plot of NHR for mortality. (C) Calibration plot of NHR for sICH. [file Image_1.TIF]

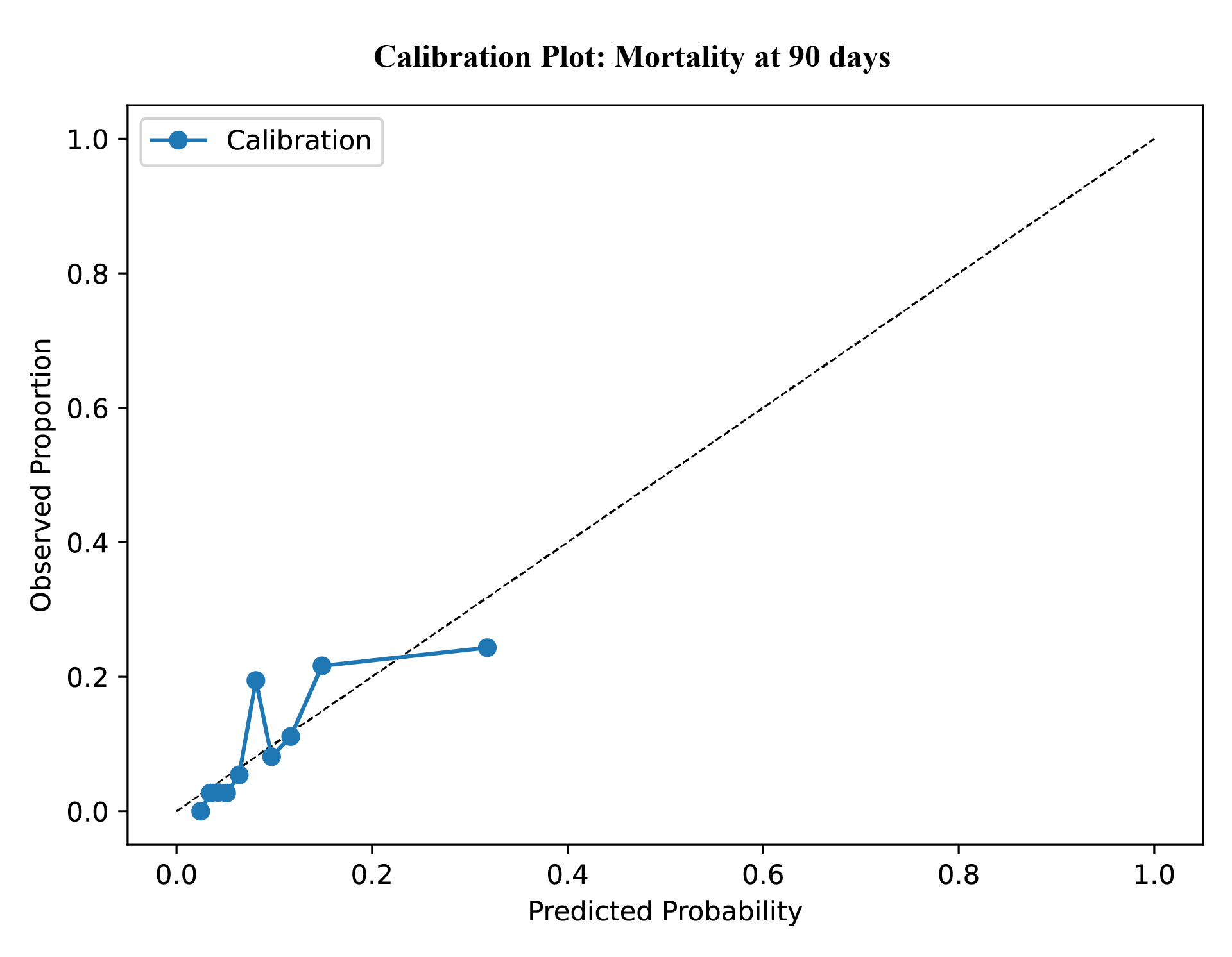

Supplement: Supplementary file 2 [file Image_2.TIF]

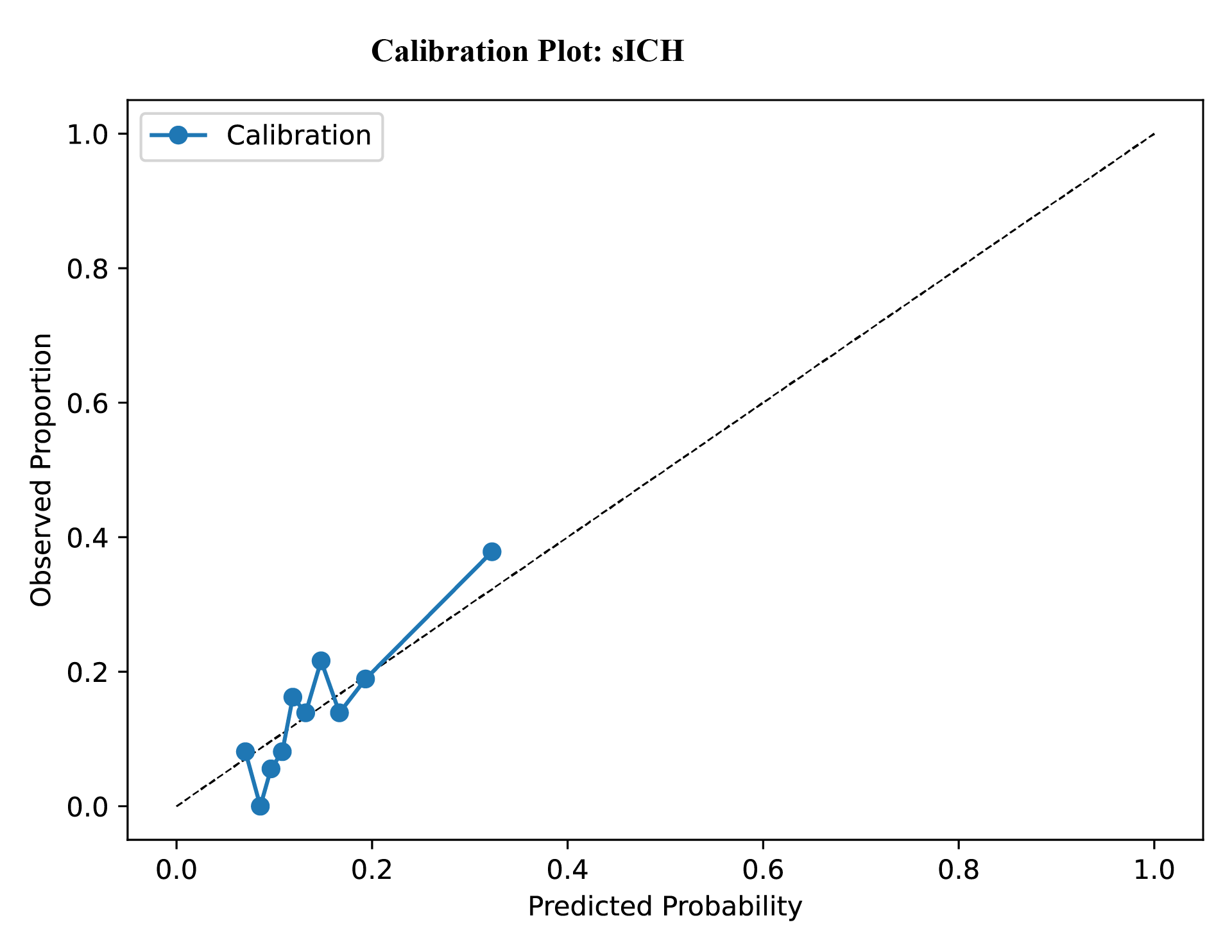

Supplement: Supplementary file 3 [file Image_3.TIF]
