# Supplementary material for: Neutrophil-to–high-density lipoprotein cholesterol ratio as a predictor of outcomes after successful endovascular reperfusion in acute ischemic stroke
Source: Front Neurol. 2026 Apr 15;17:1800774. doi: 10.3389/fneur.2026.1800774 (PMC13125049; doi:10.3389/fneur.2026.1800774)
Supplement: Supplementary file 4 [file Table_1.DOCX]

Supplementary Table S1: variance inflation factor detection in models.

|  |  | **VIF** | | **VIF** | **VIF** |
| --- | --- | --- | --- | --- | --- |
| **Model** |  | | **Poor outcome** | **Mortality**  **at 90 days** | **sICH** |
| Core-adjust model | NHR | | 2.27 | 2.27 | 2.27 |
| Core-adjust model | Age | | 12.60 | 12.60 | 12.60 |
| Core-adjust model | Sex | | 3.42 | 3.42 | 3.42 |
| Core-adjust model | NIHSS on admission | | 3.41 | 3.41 | 3.41 |
| Core-adjust model | ASPECTS | | 10.01 | 10.01 | 10.01 |
| Core-adjust model | Time from onset to groin puncture | | 3.08 | 3.08 | 3.08 |
| Expanded model | NHR | | 2.45 | 2.45 | 2.45 |
| Expanded model | Age | | 24.91 | 24.91 | 24.91 |
| Expanded model | Sex | | 3.85 | 3.85 | 3.85 |
| Expanded model | NIHSS on admission | | 4.15 | 4.15 | 4.15 |
| Expanded model | ASPECTS | | 11.91 | 11.91 | 11.91 |
| Expanded model | Anterior circulation infarction | | 6.09 | 6.09 | 6.09 |
| Expanded model | Collateral Circulation status | | 7.16 | 7.16 | 7.16 |
| Expanded model | History of stroke | | 1.33 | 1.33 | 1.33 |
| Expanded model | Hypertension | | 3.91 | 3.91 | 3.91 |
| Expanded model | Diabetes | | 1.56 | 1.56 | 1.56 |
| Expanded model | Hyperlipidemia | | 1.75 | 1.75 | 1.75 |
| Expanded model | Coronary artery disease | | 1.17 | 1.17 | 1.17 |
| Expanded model | Atrial fibrillation | | 1.86 | 1.86 | 1.86 |
| Expanded model | Intravenous thrombolysis | | 1.30 | 1.30 | 1.30 |
| Expanded model | Pneumonia | | 1.58 | 1.58 | 1.58 |
| Expanded model | Time from onset to admission | | 96.16 | 96.16 | 96.16 |
| Expanded model | Time from onset to image | | 118.41 | 118.41 | 118.41 |
| Expanded model | Time from onset to groin puncture | | 26.30 | 26.30 | 26.30 |
| Expanded model | Number of passes >3 times | | 1.89 | 1.89 | 1.89 |
| Expanded model | Arterial thrombolysis | | 1.30 | 1.30 | 1.30 |
| Expanded model | Balloon dilatation | | 2.86 | 2.86 | 2.86 |
| Expanded model | Stent implantation | | 1.58 | 1.58 | 1.58 |
| Expanded model | Stent removal | | 3.59 | 3.59 | 3.59 |
| Expanded model | Tirofiban treatment | | 3.88 | 3.88 | 3.88 |

Abbreviations: ASPECTS, Alberta Stroke Program Early Computed Tomography Score; NHR, Neutrophil-to–High-Density Lipoprotein Cholesterol Ratio; NIHSS, National Institutes of Health Stroke Scale. VIF, Variance Inflation Factor.
