# Supplementary material for: Neutrophil-to–high-density lipoprotein cholesterol ratio as a predictor of outcomes after successful endovascular reperfusion in acute ischemic stroke
Source: Front Neurol. 2026 Apr 15;17:1800774. doi: 10.3389/fneur.2026.1800774 (PMC13125049; doi:10.3389/fneur.2026.1800774)
Supplement: Supplementary file 5 [file Table_2.DOCX]

Supplementary Table S2. Exploratory NHR cut-off values

| Clinical outcome | Cutoff | Sensitivity | Specificity |
| --- | --- | --- | --- |
| **Poor outcome at 90days** | 5.80 | 0.68 | 0.69 |
| **Mortality at 90 days** | 8.62 | 0.44 | 0.83 |
| **sICH** | 7.31 | 0.62 | 0.73 |

Abbreviations:

NHR, Neutrophil-to–High-Density Lipoprotein Cholesterol Ratio;

sICH, Symptomatic intracranial hemorrhage.
